# Rs3212986 polymorphism, a possible biomarker to predict smoking‐related lung cancer, alters DNA repair capacity via regulating *ERCC1* expression

**DOI:** 10.1002/cam4.1842

**Published:** 2018-11-19

**Authors:** Tao Yu, Ping Xue, Su Cui, Liang Zhang, Guopei Zhang, Mingyang Xiao, Xiao Zheng, Qianye Zhang, Yuan Cai, Cuihong Jin, Jinghua Yang, Shengwen Wu, Xiaobo Lu

**Affiliations:** ^1^ Department of Toxicology, School of Public Health China Medical University Shenyang China; ^2^ Department of Thoracic Surgery Ward 2 The First Hospital of China Medical University Shenyang China; ^3^ Department of Thoracic Surgery Liaoning Cancer Hospital & Institute Shenyang China

**Keywords:** *CD3EAP*, DNA repair capacity, *ERCC1*C8092A (rs3212986), lung cancer, polymorphism in 3'UTR

## Abstract

Single nucleotide polymorphisms (SNPs) in 3′UTR of key DNA repair enzyme genes are associated with inter‐individual differences of DNA repair capacity (DRC) and susceptibility to a variety of human malignancies such as lung cancer. In this study, seven candidate SNPs in 3′UTR of DRC‐related genes including *ERCC1* (rs3212986, rs2336219, and rs735482), *OGG1* (rs1052133), *MLH3* (rs108621), *CD3EAP* (rs1007616), and *PPP1R13L* (rs6966) were analyzed in 300 lung cancer patients and controls from the northeast of China. Furthermore, we introduced *ERCC1* (CDS+3′UTR) or *CD3EAP* (CDS) cDNA clone to transfect HEK293T and 16HBE cells. Cell viability between different genotypes of transfected cells exposed to BPDE was detected by CCK‐8 assay, while DNA damage was visualized using γH2AX immunofluorescence and the modified comet assay. We found that minor A‐allele of rs3212986 could reflect a linkage with increasing risk of NSCLC. Compared with CC genotype, AA genotype of *ERCC1* rs3212986 was a high‐risk factor for NSCLC (OR = 3.246; 95%CI: 1.375‐7.663). Particularly stratified by smoking status in cases and controls, A allele of *ERCC1* rs3212986 also exhibited an enhanced risk to develop lung cancer in smokers only (*P* < 0.05). Interestingly, reduced repair efficiency of DNA damage was observed in 293T *ERCC1*(AA) and 16HBE *ERCC1*(AA), while no significant difference was appeared in two genotypes of *CD3EAP* (3′ adjacent gene of *ERCC1*) overexpressed cells. Our findings suggest that rs3212986 polymorphism in 3ʹUTR of *ERCC1* overlapped with *CD3EAP* may affect the repair of the damage induced by BPDE mainly via regulating *ERCC1* expression and become a potential biomarker to predict smoking‐related lung cancer.

## 1 INTRODUCTION

Lung cancer has high morbidity and mortality and becomes the leading cause of cancer death worldwide. As the most common malignant tumors in developing countries, nearly 85% of lung cancer cases are diagnosed as nonsmall cell lung cancer (NSCLC), which is mainly classified into adenocarcinoma (AC) and squamous cell carcinoma, and have various and complex pathogenesis.^1^, ^2^, ^3^ Many recent studies indicated that accumulated DNA damage caused by environmental carcinogen exposure and reduced individual DNA repair efficiency is associated with an increased risk of NSCLC development.^4^, ^5^, ^6^, ^7^

DNA repair system can maintain genome stability and reduce cancer risk by removal of diversified DNA damages via three DNA excision repair pathways, namely nucleotide excision repair (NER), base excision repair, (BER) and mismatch repair (MMR). Previous studies reported that the polymorphisms of DNA repair genes, especially those in DNA excision repair pathways involving in the restoration of DNA adducts after exposure to UV light or benzo[a]pyren ediol epoxide (BPDE, a metabolite of benzo[a]pyrene), have observably linked with the occurrence and development of lung cancer.^8^, ^9^, ^10^, ^11^ *ERCC1* (excision repair cross‐complementation group 1), *OGG1* (8‐oxoguanine DNA glycosylase), and *MLH3* (mutL homolog 3) are three key rate‐limiting enzymes involving in NER, BER, and MMR pathways, respectively. Single‐nucleotide polymorphisms (SNPs) in these genes are associated with the risk of cancers mainly due to the alerted DNA repair activity of respective enzymes.^12^, ^13^, ^14^, ^15^ However, previous epidemiological investigation and genome‐wide association studies usually focus on the significance of polymorphisms in open reading frame (ORF), while the supporting data of “regulatory SNPs” on noncoding regions especially 3ʹUTR and its relation with lung cancer have been barely reported so far. In eukaryotes, 3ʹUTR plays an important role in cellular location and mRNA regulation of gene expression. Mutations in 3ʹUTR of key DNA repair enzyme genes can contribute to interindividual differences in DNA repair efficiency, which has a crucial role in hereditary susceptibility to cancer risk and chemotherapy resistance.^16^ In our previous study, two SNPs in *ERCC1* were investigated used HPLC and modified comet assay to evaluate the individual capacity of repairing BPDE‐DNA adduct from 117 randomly selected healthy participants. The conclusion indicated that the minor A allele in rs3212986, which was located at 3ʹUTR (3ʹ untranslated region) of *ERCC1* and overlapped with coding region of *CD3EAP* (CD3e molecule, epsilon‐associated protein, antisense *ERCC1*), resulted in a diminished capacity of repairing BPDE‐DNA adducts.^17^ *CD3EAP*, known as a nucleoprotein and reverse complementary to *ERCC1*, may serve as a component of the RNA polymerase I transcription complex implicating in cell proliferation^18^ and coordinately overlaps with *PPP1R13L* (protein phosphatase 1, regulatory subunit 13 like, RelA‐associated inhibitor), which is an inhibitor of p53 and NF‐κB affecting the regulation of apoptotic pathway and inflammatory response.^19^, ^20^, ^21^ It is reported that the polymorphisms of overlapping gene *ERCC1, CD3EAP,* and *PPP1R13L* on the chromosomal region 19q13.3 were associated with individual DNA repair capacity (DRC) and lung cancer susceptibility,^22^ which suggested that this exceptional overlapping structure of three genes may have potential functions in carcinogenesis. However, the functional properties based on neighboring SNPs in haplotypes of crucial DNA repair enzymes and DRC‐related proteins as well as the predictive value and the related mechanism of DNA excision repair gene polymorphisms in 3ʹUTR are still largely uncertainty.

In this case‐control study, seven candidate SNPs in 3ʹUTR of DRC‐related genes, including *ERCC1* (rs3212986, rs2336219, and rs735482), *OGG1* (rs1052133), *MLH3* (rs108621), *CD3EAP* (rs1007616), and *PPP1R13L* (rs6966), were analyzed in well‐characterized series of 300 lung cancer patients matched with 300 healthy controls from the northeast of China to prospectively evaluate the associations between DRC‐related polymorphisms in 3ʹUTR and the risk of lung tumorigenesis. Furthermore, the effects of the target SNPs on the capacity of repairing BPDE‐DNA adducts were also investigated using an in vitro transfected cell model. Data from the presented study further indicated that rs3212986 polymorphism in 3ʹUTR of *ERCC1* overlapped with *CD3EAP* may affect the repair of the damage induced by BPDE mainly via regulating *ERCC1* expression and become a potential biomarker to predict smoking‐related lung cancer.

## 2 MATERIAL AND METHODS

### 2.1 Study subjects

In this study, we recruited 300 patients suffering from lung cancer from the First Affiliated Hospital of China Medical University as our cases. The cases were diagnosed from 2013 to 2015 by the local authorized diagnosing pathologists. Control subjects were healthy volunteers who visited the hospital for physical examination. The controls had no history of cancers and were matched to the cases by age (±3 years), gender, and ethnicity and recruited in the same region and period. The protocol and consent form were approved by the Institutional Review Board of China Medical University. All activities involving human subjects were done under full compliance with government policies and the Helsinki Declaration. Informed consent was obtained from each of the participants after a detailed explanation of the nature and possible consequences of the study. Each participant donated 5 mL venous blood, their demographic data were recorded in questionnaires in detail, and the study participant consents were obtained prior to the study. Those who smoked at least one cigarette per day for more than 1 year were considered regular smokers.

### 2.2 DNA extraction and TaqMan^®^ SNP Genotyping Assays

Genomic DNA was extracted as our published method.^17^ *ERCC1*(rs3212986, C>A, assay ID C_2532948_10; rs735482, A>C, assay ID C_341729_10; rs2336219, G>A, assay ID C_16204465_10), *MLH3*(rs108621, T>C, assay ID C_2178406_10), *OGG1*(rs1052133, G>C, assay ID C_3095552_1), *PPP1R13L*(rs6966, T>A, assay ID C_2615637_10), and *CD3EAP* (rs1007616, C>T, assay ID AH6RTHI) were purchased from ABI Company (ABI, US, Stagapore) and analyzed by TaqMan^®^ genotyping on a LightCycler 480 Real‐time PCR system (Roche, Foster City, CA, USA). All PCR reagents were purchased from Roche Company (Roche).

The PCRs were performed in a 20 μL reaction mixture: 10 μL of probe Mix, 5 μL (1×) of assay mix, and 2 μL of DNA (25 ng/μL). The PCR included an initial step at 95°C for 10 minutes; 40 cycles of denaturation at 95°C for 10 second, extension at 60°C for 1 minutes and 72°C for 1 second; and at the end, cooling at 40°C for 30 second.

### 2.3 Chemicals and plasmids construction

Benzo[a]pyrene 7, 8‐diol 9, 10‐epoxide was purchased from National Cancer Institute Chemical Carcinogen Repository (Midwest Research Institute, Kansas City, MO, USA).

Plasmids expressing the ORFs of rs3212986 C or A allele in *ERCC1* [pLenti‐EGFP‐CMV‐ERCC1 (CDS+3′UTR)] or *CD3EAP* [pLenti‐mcherry‐CMV‐CD3EAP (CDS)] and the firefly luciferase reporter plasmid were constructed by Obio Technology CO (Shanghai, China).

### 2.4 Cell culture and treatment

The immortalized 16HBE cell line, kindly provided by Prof. Wen Chen (Sun Yat‐Sen University, China), was cultured in MEM (Hyclone, Waltham USA). HEK293T and A549 cells were purchased from the Cell Bank of the Shanghai Institute of Biochemistry and Cell Biology, Chinese Academy of Sciences, and cultured in DMEM/F‐12 (Hyclone) and DMEM (Hyclone), respectively. Both cells were supplemented with 10% fetal bovine serum (Hyclone) and maintained at 37°C, 5% CO_2_ in a humidified incubator.

All experiments were conducted using the cells at a logarithmic stage of growth curve. In accordance with the following treatments before experiments of cell viability and DNA repair assay, cells were seeded and 24 hours later the plasmids were introduced to the HEK293T or 16HBE with Lipofectamine™ 3000 Reagent (Invitrogen, Carlsbad, CA, USA) according to the manufacturer’s recommendation, and the exponentially growing transfected cells were exposed to BPDE after 24 hours.

### 2.5 Cell Counting Kit‐8 assay

Cell viability was evaluated using Cell Counting Kit‐8 (Dojindo, Japan). The transfected HEK293T cells were incubated with various concentrations of BPDE (0.1, 0.5, 1, 1.5, 2, 4, 8, 16 µmol/L) for 24 hours and an additional 24 hours for DNA repair after treatment. Then, 10 µL of the Cell Counting Kit‐8 (CCK‐8) reagent was added to each well and incubated for another 3.5 hours. The absorbance (value) at 450 nm was measured using a scanning microplate reader (Biotek, Winooski, VT, USA).

### 2.6 Comet assay

Damage and repair of DNA adducts was assessed by alkaline comet assay with some modifications.^18^ Cells were harvested for the following procedures: precoated microscope slides were covered by dropping 100 µL 1% normal melting point agarose and 100 µL gel mixture of cells and 1% low melting point agarose. Gel slides were soaked in cell lysis solution (100 mmol/L disodium EDTA, 2.5 M NaCl, 10 mM Tris‐HCl pH 10.5, adding 1% Triton X‐100 before using) for 60‐90 minutes and immersed in the electrophoresis solution (1 mol/L disodium EDTA, 300 mmol/L NaOH, pH = 13) for 30 minute to denature DNA. After electrophoresis at 20 V (100 mA) for 20 minute, glasses were rinsed three times with 0.4 mol/L Tris‐HCl (pH 7.5). Due to the background color of the transfected cells, propidium Iodide or Goldview (green fluorescence) was used for staining. Approximately 100‐200 comets were analyzed per experiment. Results were expressed as mean tail moment.

### 2.7 Immunofluorescence microscopy

γH2AX proteins are concentrated at the damaged DNA strand and can be visualized as foci by immunofluorescence. Cells were fixed in 4% paraformaldehyde and permeabilized with 0.5% Triton X‐100/PBS. Coverslips were blocked with 1% BSA for 30 minutes and then incubated overnight with γH2AX‐antibody (Abcam, Cambridge, UK, ab2893, 1:200). Cells were incubated with secondary antibody Alexa Fluor 488 or 594 conjugated goat anti‐rabbit IgG (proteintech, 1:150) for 1 hour at room temperature. Next, cells were washed with PBS and incubated with DAPI (Beyotime, Shanghai, China) for nuclei staining. Foci on each slide were visualized by fluorescence microscope, and cells containing at least 10 foci were denoted as positive controls. At least 200 cells per slide were scored.

### 2.8 *ERCC1* mRNA secondary structure predictions and optimal free energy calculations

Secondary structures for either rs3212986 C allele or A allele of *ERCC1* mRNA sequences and the minimum optimal free energies were predicted by using RNA structure 5.3 software (Mathew Lab, Rochester, NY, USA). The predictions and calculations were performed according to the instructions and the default setting of the program.

### 2.9 Statistical analysis

All data obtained were analyzed by IBM SPSS 19.0 (IBM Company, Armonk, NY, USA) and GraphPad Prism 5.0 software (GraphPad Software Inc, San Diego, CA, USA). The Hardy‐Weinberg equilibrium for three SNPs in *ERCC1*, four SNPs in overlapping regions of *CD3EAP* and *ERCC1*, and the level of linkage disequilibrium (LD) were analyzed by Haploview 4.2 Software （Broad Institute， Cambridge, UK). Whether rs3212986 variants could affect *ERCC1* mRNA secondary structure was predicted by RNA structure 5.3 software. The chi‐square test and logistic regression were used to evaluate the association between genetic polymorphisms and the risk of lung cancer. *t* Test was used to estimate the differences among the groups. A two‐tailed *P*‐value <0.05 was considered statistically significant.

## 3 RESULTS

### 3.1 Genetic polymorphisms in *ERCC1, OGG1*,* MLH3, PPP1R13L,* and *CD3EAP* and the risk of lung cancer

We initially screened the SNPs of selected genes to predict the risk of lung cancer using NCBI database combining with the minor allele frequency (MAF) (Table S1). According to MAF and sample size of our current study, we chose the following target SNPs: *ERCC1 (*rs3212986, rs2336219, and rs735482), *MLH3* (rs108621), *OGG1 (*rs1052133), *PPP1R13L (*rs6966), and *CD3EAP (*rs1007616).

The study population consisted of 300 lung cancer cases and 300 cancer‐free controls, which had the same composition ratio of 188 men and 112 women (Table S2). The lung cancer cases’ ages ranged from 35 to 84 years (mean, 59.54 years and median, 60 years). Controls were characterized by a median age of 59 (range: 33‐85 and mean, 60.04 years). *ERCC1* rs3212986 AA genotype revealed a higher risk of lung cancer compared to CC genotype (OR = 2.061, 95%CI: 1.057‐4.017) (Table 1). After stratifying by gender (Table 2), rs3212986 AA genotype was also at higher risk of lung cancer among men (OR = 3.246, 95%CI: 1.375‐7.663). No association was observed in female. In addition, the *OGG1* rs1052133 CC genotype (OR = 2.588, 95%CI: 1.035‐6.474) exhibited a higher risk of lung cancer development in females. The analysis of age stratification showed that *ERCC1* rs735482 AC genotype had a protective role on lung cancer (OR = 0.560, 95%CI: 0.336‐0.934) (Table 3).

**Table 1:** Genotypes for genetic polymorphisms in *ERCC1*,* OGG1*,* MLH3*,* PPP1R13L,* and *CD3EAP* and their effects on the risk of lung cancer

SNPs	Cases		Control		OR (95%CI)	χ^2^	*P* [Link]
	n	%	n	%			
*ERCC1* rs3212986						5.344	0.069
CC	152	50.7	174	58.0	1.000		
CA	121	40.3	111	37.0	1.248 (0.891‐1.748)	1.658	0.198
AA	27	9.0	15	5.0	2.061 (1.057‐4.017)	4.645	**0.031**
*ERCC1* rs735482						1.387	0.500
AA	92	30.7	79	26.3	1.000		
AC	150	50.0	160	53.3	0.805 (0.554‐1.170)	1.292	0.256
CC	58	19.3	61	20.4	0.816 (0.511‐1.305)	0.720	0.369
*ERCC1* rs2336219						0.885	0.642
GG	92	30.7	83	27.7	1.000		
GA	151	50.3	162	54.0	0.841 (0.581‐1.218)	0.841	0.359
AA	57	19.0	55	18.3	0.935 (0.582‐1.503)	0.077	0.781
*MLH3* rs108621						1.590	0.452
TT	190	63.3	201	67.0	1.000		
TC	96	32.0	90	30.0	1.128 (0.796‐1.600)	0.460	0.498
CC	14	4.7	9	3.0	1.646 (0.696‐3.891)	1.310	0.252
*OGG1* rs1052133						1.388	0.499
GG	96	32.0	106	35.3	1.000		
GC	154	51.3	153	51.0	1.111 (0.799‐1.586)	0.339	0.560
CC	50	16.7	41	13.7	1.347 (0.819‐2.213)	1.382	0.240
*PPP1R13L* rs6966						1.595	0.451
TT	80	26.7	85	28.3	1.000		
TA	145	48.3	130	43.4	1.185 (0.805‐1.744)	0.743	0.389
AA	75	25.0	85	28.3	0.938 (0.607‐1.449)	0.084	0.771
*CD3EAP* rs1007616						2.451	0.294
CC	159	53.0	178	59.4	1.000		
CT	115	38.3	100	33.3	1.287 (0.914‐1.814)	2.089	0.148
TT	26	86.7	22	7.3	1.323 (0.721‐2.427)	0.821	0.365

*P*‐value <0.05 was considered statistically significant (in bold).

Pearson chi‐square test for difference in distributions between the case and control groups.

**Table 2:** The relationship between gender status and genetic polymorphisms in five selected genes

Groups	Male		OR (95% CI)	Female		OR (95% CI)
	Cases (%)	Controls (%)		Cases (%)	Cases (%)	
*ERCC1* rs3212986						
CC	93 (49.5)	115 (61.2)	1.000	59 (52.7)	59 (52.7)	1.000
CA	74 (39.4)	65 (34.6)	1.408 (0.915‐2.166)	47 (42.0)	46 (41.1)	1.022 (0.593‐1.760)
AA	21 (11.1)	8 (4.2)	3.246 (1.375‐7.663)[Link]	6 (5.3)	7 (6.2)	0.857 (0.272‐2.703)
*ERCC1* rs735482						
AA	64 (34.0)	45 (23.9)	1.000	28 (25.0)	34 (30.4)	1.000
AC	89 (47.3)	100 (53.2)	0.626 (0.389‐1.008)	61 (54.5)	60 (53.6)	1.235 (0.668‐2.282)
CC	35 (18.6)	43 (22.9)	0.572 (0.318‐1.029)	23 (20.5)	18 (16.0)	1.522 (0.701‐3.433)
*ERCC1* rs2336219						
GG	64 (34.0)	47 (25.0)	1.000	28 (25.0)	36 (32.1)	1.000
GA	90 (47.9)	103 (54.8)	0.642 (0.401‐1.208)	61 (54.5)	59 (52.7)	1.329 (0.722‐2.446)
AA	34 (18.1)	38 (20.2)	0.657 (0362‐1.193)	23 (20.5)	17 (15.2)	1.739 (0.783‐3.864)
*MLH3* rs108621						
TT	123 (65.4)	125 (66.5)	1.000	67 (59.8)	76 (67.9)	1.000
TC	55 (29.3)	58 (30.9)	0.964 (0.618‐1.504)	41 (36.6)	32 (28.6)	1.453 (0.824‐2.563)
CC	10 (5.3)	5 (2.7)	2.033 (0.675‐6.118)	4 (6.6)	4 (3.5)	1.134 (0.273‐4.713)
*OGG1* rs1052133						
GG	62 (33.0)	62 (33.0)	1.000	34 (30.4)	34 (39.3)	1.000
GC	94 (50.0)	94 (50.0)	1.000 (0.635‐1.574)	60 (53.6)	59 (52.7)	1.316 (0.741‐2.336)
CC	32 (17.0)	32 (17.0)	1.000 (0.547‐1.828)	18 (16.1)	9 (8.0)	2.588 (1.035‐6.474)[Link]
*PPP1R13L* rs6966						
TT	51 (27.1)	54 (28.7)	1.000	29 (25.9)	31 (27.7)	1.000
TA	89 (47.3)	80 (42.6)	1.178 (0.723‐1.918)	56 (50.0)	50 (44.6)	1.197 (0.635‐2.257)
AA	48 (25.6)	54 (28.7)	0.941 (0.545‐1.624)	27 (24.1)	31 (27.7)	0.931 (0.452‐1.918)
*CD3EAP* rs1007616						
CC	102 (54.3)	116 (61.7)	1.000	57 (50.9)	62 (55.3)	1.000
CT	66 (35.1)	61 (32.4)	1.230 (0.794‐1.907)	49 (43.8)	39 (34.8)	1.367 (0.786‐2.377)
TT	20 (10.6)	11 (5.9)	2.068 (0.946‐4.521)	6 (5.3)	11 (9.9)	0.593 (0.206‐1.709)

χ^2^ = 7.824 *P* = 0.005.

χ^2^ = 4.273 *P* = 0.039.

**Table 3:** The relationship between age status and genetic polymorphisms in five selected genes

Groups	Age < 60 (y)		OR (95% CI)	Age ≥ 60 (y)		OR (95% CI)
	Cases (%)	Controls (%)		Cases (%)	Controls (%)	
*ERCC1* rs3212986						
CC	67 (45.0)	85 (53.8)	1.000	85 (56.3)	89 (62.7)	1.000
CA	68 (45.6)	63 (39.9)	1.369 (0.857‐2.189)	53 (35.1)	48 (33.8)	1.248 (0.891‐1.748)
AA	14 (9.4)	10 (6.3)	1.776 (0.742‐4.250)	13 (8.6)	5 (3.5)	2.722 (0.931‐7.964)
*ERCC1* rs735482						
AA	53 (35.6)	40 (25.3)	1.000	39 (25.8)	39 (27.5)	1.000
AC	72 (48.3)	97 (61.4)	0.560 (0.336‐0.934)[Link]	78 (51.7)	63 (44.4)	1.238 (0.711‐2.155)
CC	24 (16.1)	21 (13.3)	0.863 (0.422‐1.764)	34 (22.5)	40 (28.1)	0.850 (0.449‐1.607)
*ERCC1* rs2336219						
GG	53 (35.6)	42 (26.6)	1.000	39 (25.8)	41 (28.9)	1.000
GA	74 (49.7)	95 (60.1)	0.617 (0.372‐1.024)	77 (51.0)	67 (47.2)	1.208 (0.699‐2.088)
AA	22 (14.8)	21 (13.3)	0.830 (0.403‐1.709)	35 (23.2)	34 (23.9)	1.082 (0.568‐2.061)
*MLH3* rs108621						
TT	96 (64.4)	100 (63.3)	1.000	94 (62.3)	101 (71.1)	1.000
TC	46 (30.9)	55 (34.8)	0.871 (0.538‐1.410)	50 (33.1)	35 (24.6)	1.535 (0.917‐2.570)
CC	7 (4.7)	3 (1.9)	2.431 (0.611‐9.673)	7 (4.6)	6 (4.3)	1.254 (0.407‐3.865)
*OGG1* rs1052133						
GG	41 (27.5)	61 (38.6)	1.000	55 (36.4)	45 (31.7)	1.000
GC	85 (57.0)	78 (49.4)	1.621 (0.982‐2.676)	69 (45.7)	75 (52.8)	0.753 (0.451‐1.256)
CC	23 (15.4)	19 (12.0)	1.801 (0.872‐3.719)	27 (17.9)	22 (15.5)	1.004 (0.505‐1.996)
*PPP1R13L* rs6966						
TT	37 (24.8)	43 (27.2)	1.000	43 (28.5)	42 (29.6)	1.000
TA	70 (47.0)	68 (43.1)	1.196 (0.689‐2.077)	75 (49.6)	62 (43.7)	1.182 (0.687‐2.032)
AA	42 (28.2)	47 (29.7)	1.039 (0.567‐1.902)	33 (21.9)	38 (26.7)	0.848 (0.451‐1.594)
*CD3EAP* rs1007616						
CC	83 (55.7)	96 (60.8)	1.000	76 (50.3)	82 (57.7)	1.000
CT	53 (35.6)	55 (34.8)	1.115 (0.691‐1.798)	62 (41.1)	45 (31.7)	1.487 (0.906‐2.438)
TT	13 (8.7)	7 (4.4)	2.148 (0.819‐5.636)	13 (8.6)	15 (10.6)	0.935 (0.418‐2.093)

χ^2^ = 4.976, *P* = 0.026.

### 3.2 Association between the risk of lung cancer and *ERCC1* rs3212986 under smoking status

Cancers are likely caused by the interactions among environmental exposure, genetic polymorphisms, and lifestyle behaviors,^23^ such as tobacco exposure is one of the major environmental high‐risk factors to develop lung cancer.^24^ We further matched 186 lung cancer cases with 186 controls (Table S3) that smoking status was clearly identified, to further explore whether there is a potential interaction between rs3212986 and the risk of lung cancer under tobacco smoking exposure. Table 4 shows that for smokers, the A allele (AA and CA + AA genotypes) of rs3212986 exhibited an enhanced risk to develop lung cancer (*P* < 0.05). No association among nonsmokers was found in the presented study.

**Table 4:** The relationship between smoking status and genetic polymorphisms in *ERCC1*

Groups	Smoking		OR (95% CI)	No smoking		OR (95% CI)
	Cases	Controls		Cases	Controls	
*ERCC1* rs3212986						
CC	40	54	1.000	55	60	1.000
CA	28	22	1.718 (0.860‐3.433)	45	38	1.292 (0.734‐2.276)
AA	13	5	3.510 (1.157‐10.645)[Link]	5	7	0.779 (0.234‐2.599)
CA + AA	41	27	2.050 (1.086‐3.868)[Link]	50	45	1.212 (0.703‐2.089)

χ^2^ = 5.335, *P* = 0.021.

χ^2^ = 4.967, *P* = 0.026.

### 3.3 Effects of haplotypes in *ERCC1* and *CD3EAP* on the risk of lung cancer

**Figure 1:**
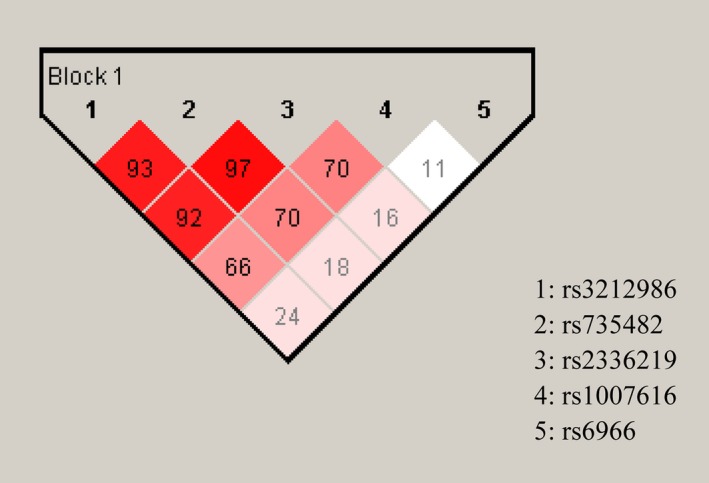
Analysis of haplotype blocks in the overlapping regions of ERCC1, CD3EAP, and PPP1R13L

The association between inferred haplotypes and lung cancer risk is shown in Table 5. Three SNPs (rs3212986, rs735482, and rs2336219) of *ERCC1* in 3ʹUTR were differentiated into four haplotypes on account of significant linkage disequilibrium (Figure 1). Compared with CAG haplotype as the reference, CCG haplotype was presented a protective effect against lung cancer risk (OR = 0.291, 95%CI: 0.062‐0.769), while AAG haplotype including the minor A allele of *ERCC1*rs3212986 was noted for a trend toward increased risk of lung cancer (OR = 1.350, 95%CI: 0.989‐1.841) with critical *P* value. In addition, an integrated haplotype analysis of 5 SNPs in *ERCC1*,* CD3EAP,* and *PPP1R13L* on 19q13 was performed to explore higher LD with the cancer‐related SNP. However, four SNPs (*CD3EAP* rs1007616, *ERCC1*rs3212986, *ERCC1*rs735482, and *ERCC1* rs2336219) were actually accepted into haplotype blocks analysis besides *PPP1R13L* rs6966, which was in weaker LD with others (Figure 1). Compared with wild‐type haplotype block, haplotype block CCAC including both the minor C allele of *ERCC1* rs735482 and A allele of *ERCC1* rs2336219 (OR = 14.323, 95%CI: 6.503‐31.547); haplotype block AAGC including the minor A allele of *ERCC1* rs3212986 (OR = 17.692, 95%CI: 7.909‐39.576); haplotype block CAGT including the minor T allele of *CD3EAP* rs1007616 (OR = 22.646, 95%CI: 10.051‐51.026); haplotype block AAGT including both the minor A allele of *ERCC1* rs3212986 and T allele of *CD3EAP* rs1007616 (OR = 5.000, 95%CI: 1.562‐16.005) were significantly associated with the high risk of lung cancer. From the above, it was implied that polymorphisms of *ERCC1* and its overlapping gene *CD3EAP* were in strong LD. Particularly, minor A allele of rs3212986 in overlapping region of two genes may be a potentially functional SNP contributing to the high risk of lung cancer and low efficiency of DNA repair.

**Table 5:** Association between the haplotypes and the risk of lung cancer

Groups	Frequency[Link]	Group		OR (95% CI)	*P* [Link]
		Cases (n)	Controls (n)		
*ERCC1* rs3212986 C>A; *ERCC1* rs735482 A>C; *ERCC1* rs2336219 G>A					
CAG	0.283	162	177	1.000	
CCA	0.435	258	264	1.068 (0.812, 1.404）	0.639
AAG	0.254	168	136	**1.350 (0.989, 1.841）**	**0.058**
**CCG**	**0.015**	**3**	**15**	**0.219 (0.062, 0.769）**	**0.010**
*ERCC1* rs3212986 C>A; *ERCC1* rs735482 A>C; *ERCC1* rs2336219 G>A; *CD3EAP* rs1007616 C>T					
CAGC	0.081	7	90	1.000	
**CCAC**	**0.402**	**254**	**228**	**14.323 (6.503**‐**31.547)**	<**0.05**
**AAGC**	**0.232**	**161**	**117**	**17.692 (7.909**‐**39.576)**	<**0.05**
**CAGT**	**0.202**	**155**	**88**	**22.646 (10.051**‐**51.026)**	<**0.05**
CCAT	0.033	4	35	1.469 (0.405‐5.333)	0.810
**AAGT**	**0.021**	**7**	**18**	**5.000 (1.562, 16.005)**	<**0.05**
CCGC	0.013	3	12	3.214 (0.731, 14.128)	0.259

*P*‐value <0.05 was considered statistically significant (in bold).

A frequency of <0.01 is not included in the Table.

The *P* value was obtained using the chi‐square test.

### 3.4 Design plasmids according to rs3213986 location and obtain transfected cells

**Figure 2:**
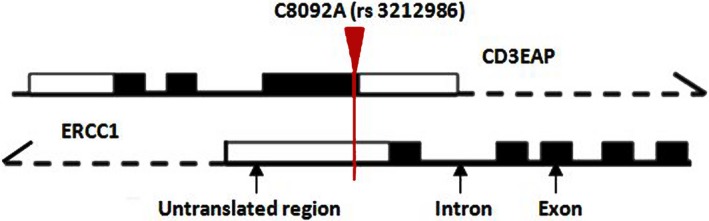
The diagram of positional relationship between ERCC1 and CD3EAP. ERCC1 C8092A (rs3213986) polymorphism located in the overlapping region of ERCC1 3′‐untranslated region and CD3EAP coding region

Rs3213986 is located in the 3′UTR of *ERCC1*, but also in the opposite orientated, adjacent coding region of *CD3EAP* (Figure 2). We first introduced full‐length *ERCC1* (CDS+3′‐UTR) or *CD3EAP* (CDS) cDNA clone containing rs3213986 CC or AA genotype. The cDNA clone of different polymorphisms transfected into the tool cells HEK293T which showed high transfection efficiency or human bronchial epithelial cells 16HBE that could better exhibit the human cell state under exogenous chemical stimulation. The obtained transfected HEK293T and 16HBE cells were designated as 293T^ERCC1/CD3EAP(CC)^ and 293T^ERCC1/CD3EAP(AA)^, or 16HBE^ERCC1/CD3EAP(CC)^ and 16HBE^ERCC1/CD3EAP(AA)^. *ERCC1*/*CD3EAP*(EV) represented the cells transfected with empty vector that not expressing *ERCC1* or *CD3EAP*, but just show colors. *ERCC1* (CDS+3′‐UTR) vector showed green fluorescence while *CD3EAP* (CDS) displayed red one. The mock and empty vectors as control expressed significantly lower *ERCC1* or *CD3EAP* protein levels than overexpressed cells.

### 3.5 Rs3212986 A allele decreased cell viability after BPDE treatment via *ERCC1*

**Figure 3:**
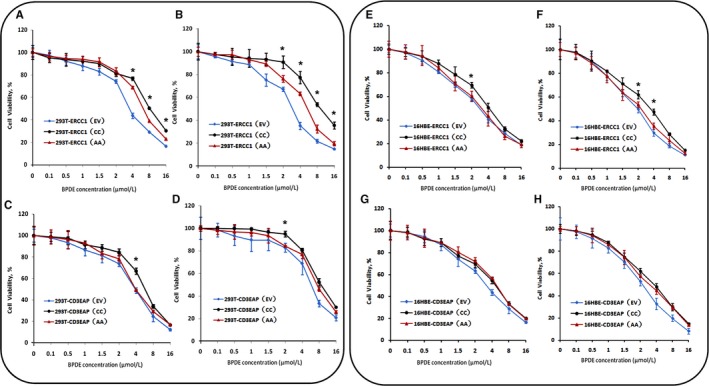
Comparison of cell viability in transfected cells after BPDE treatment. Cells survival rate of the transfected cells at 0.1, 0.5, 1, 1.5, 2, 4, 8, 16 μmol/L BPDE treatment. ERCC1 cDNA with either C allele or A allele of rs3212986 was transfected to HEK293T (A) or 16HBE (E) cells and treated with BPDE for 24 h, and another 24 h repair after 24 h treatment (B, F). CD3EAP cDNA with either C allele or A allele of rs3212986 was transfected to HEK293T (C) or 16HBE (G) cells and treated with BPDE for 24 h, and another 24 h repair after 24 h treatment (D, H). * *P* < 0.05 compared between ERCC1(CC) and ERCC1(AA), or CD3EAP(CC) and CD3EAP(AA) transfected cells

After 24 hour BPDE treatment, 293T^ERCC1(CC)^ showed more resistant to BPDE compared to 293T^ERCC1(AA)^, and the differences in survival rate between those became more pronounced after an additional 24 hour incubation(Figure 3A,B). In 16HBE transfected cells, *ERCC1* overexpression with rs3212986 CC genotype could enhance the cell resistance to BPDE. After an additional 24 hour of incubation to allow DNA repair, the survival rate of 16HBE^ERCC1(CC)^ significantly relieved at 2, 4 µmol/L BPDE treatment compared to 16HBE^ERCC1(AA)^ (Figure 3E,F). In addition, there were no statistical differences between these two genotypes of *CD3EAP* transfected 293T and 16HBE cells, except under some sensitive points (*P* < 0.05) (Figure 3C,D,G,H).

In summary, it suggested that different genotypes of rs3212986 can affect survival rates via *ERCC1* under BPDE treatment in our cell model. According to the survival curve of all transfected cells, 1, 2, and 4 µmol/L BPDE treatment were shown a distinguished alteration and selected as the target concentrations for the following experiment for DNA damage and repair.

### 3.6 A allele of rs3212986 inhibited DNA repair of BPDE‐induced damage via *ERCC1*

**Figure 4:**
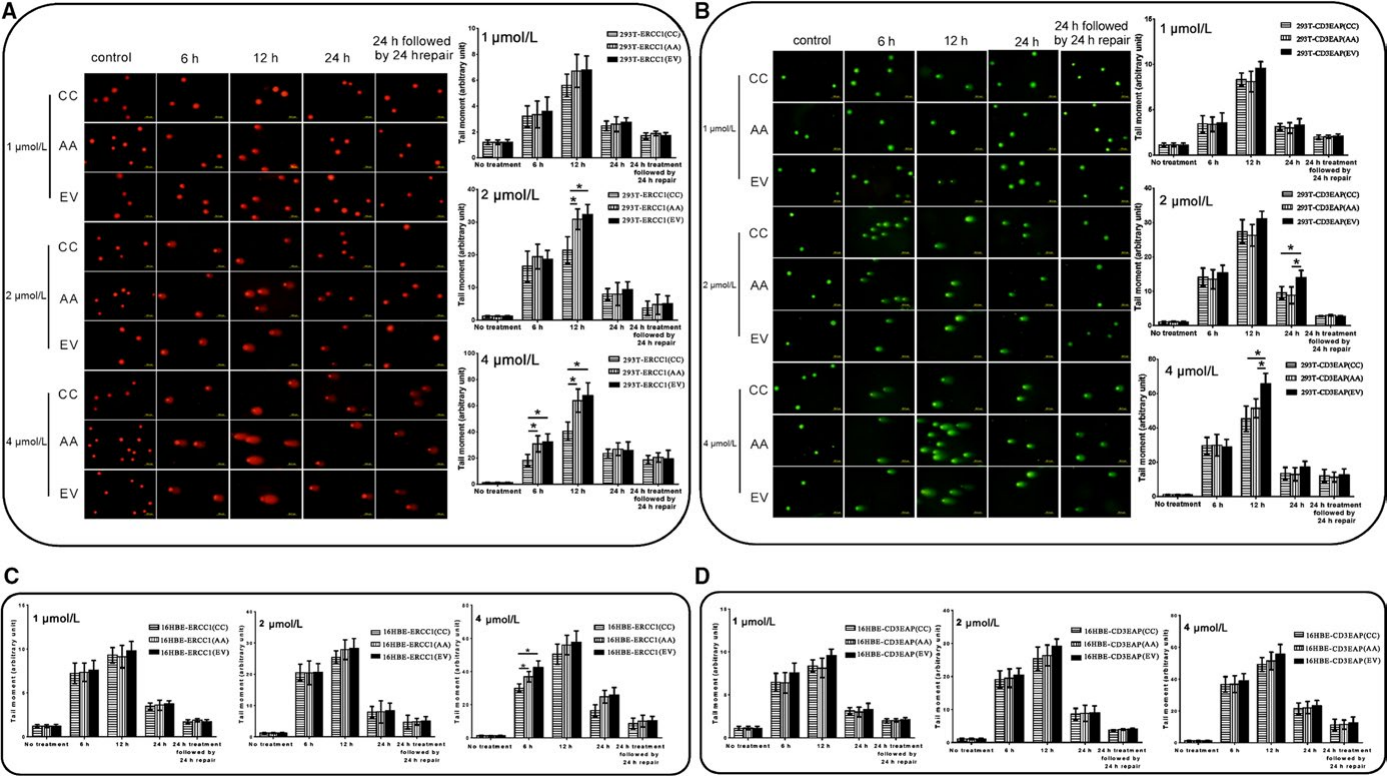
Comet assay. Comet assay of HEK293T (A,B) or 16HBE (C,D) transfected cells, either untreated (control) or exposed to 1, 2, 4 μmol/L BPDE for 6, 12, 24 h, or 24 h treatment followed by 24 h repair. The tail moment of 200 comets was analyzed for each experiment, n = 3. *P < 0.05

BPDE reacts with many biological macromolecules including DNA to cause covalent BPDE‐DNA adducts and oxidative damage.^25^ To further clarify the underlying function on DRC of rs3212986, a comet assay and immunofluorescence microscopy of γH2AX used to quantify DNA damage level induced by BPDE. For the concerning time points, the comet tail moment (OTM, as an index of DNA damage level) increased in a dose‐dependent way in the cell lines, and by additional 24 hour incubation, cell damage was recovered except for 4 µmol/L BPDE treatment. The comet tails in 293T^ERCC1(CC)^ were statistically smaller than that in 293T^ERCC1(AA)^ at 12 hour of 2 µmol/L and 6 hour, 12 hour of 4 µmol/L BPDE treatment (*P* < 0.05) (Figure 4A). No clear differences between comets in 293T^CD3EAP(CC)^ and 293T^CD3EAP(AA)^ were found. However, the longest comet tails were observed in 293T^CD3EAP(EV)^ at 24 hour of 2 µmol/L and 12 hour of 4 µmol/L BPDE treatment (*P* < 0.05) (Figure 4B).

**Figure 5:**
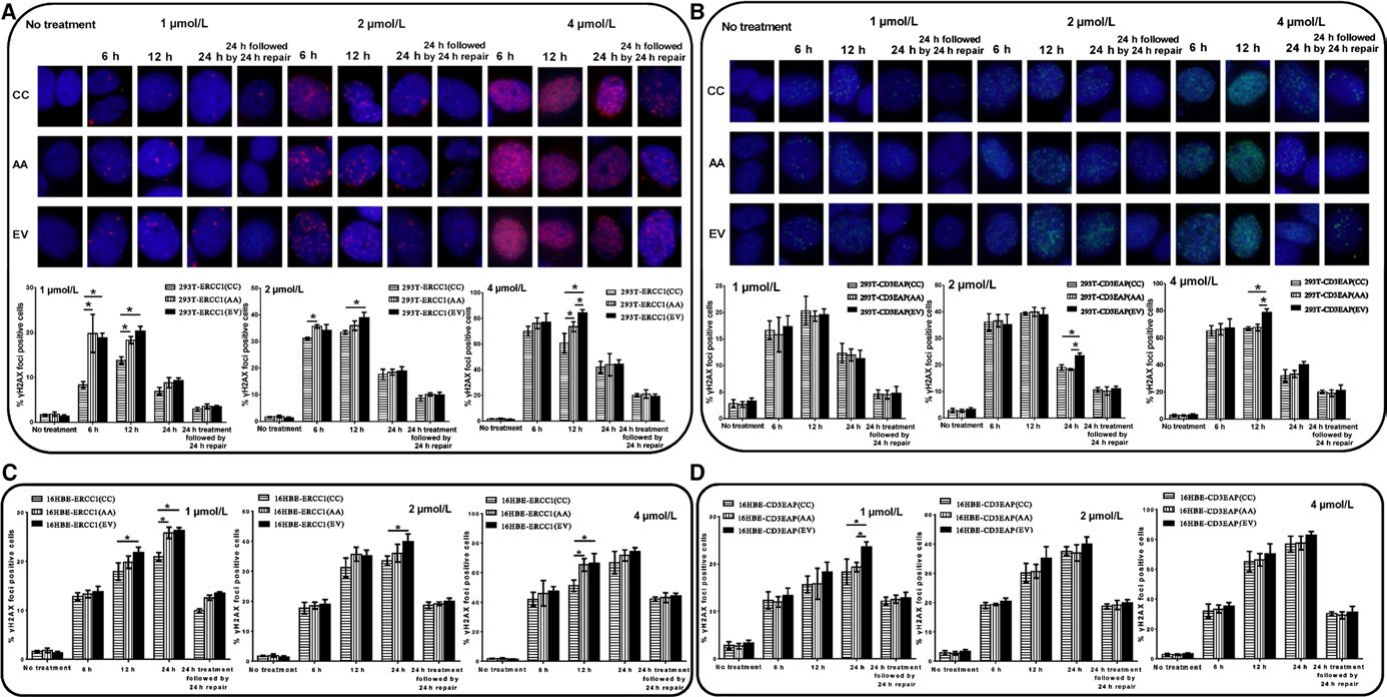
Immunofluorescence results of nuclear γH2AX foci in transfected cells in response to DNA damage. γH2AX foci of HEK293T (A,B) or 16HBE (C,D) transfected cells, either untreated (control) or exposed to 1, 2, 4 μmol/L BPDE for 6, 12, 24 h or 24 h treatment followed by 24 h repair. A cell with more than ten distinct foci in the nucleus was considered to be positive. Depicted is the average and standard deviation of three independent experiments. *P < 0.05

BPDE‐DNA adducts and DNA double‐strand breaks induced by BPDE could induce phosphorylation of H2AX serine 139 (S139).^21^ Here, we analyzed the induction of γH2AX foci by immunofluorescence response to DNA damage in cells treated with 1, 2, or 4 μmol/L BPDE. The γH2AX foci had a dose‐dependent relationship with BPDE concentrations. Between 6 and 12 hour of BPDE treatment, γH2AX foci formation was at the maximum. The 293T^ERCC1(CC)^ cells showed a better repair capacity than 293T^ERCC1(AA)^ (*P* < 0.05) (Figure 5A). Compared with the *CD3EAP* transfected HEK293T cells, the γH2AX foci percentage of 293T^CD3EAP(EV)^ was observed statistically higher at 24 hour of 2 µmol/L and 12 hour of 4 µmol/L BPDE treatment (*P* < 0.05) (Figure 5B).

For 16HBE transfected cells, *ERCC1* overexpression with rs3212986 CC genotype could enhance the DRC to BPDE (Figures 4C and 5C). In addition, the *CD3EAP* transfected 16HBE cells showed better DRC than *CD3EAP* (EV) cells after BPDE treatment (*P* < 0.05), but no clear differences between 16HBE^CD3EAP(CC)^ and 16HBE^CD3EAP(AA)^(Figures 4D and 5D). The above results further confirmed that rs3212986 mainly takes actions via affecting the function of *ERCC1*, and AA genotype of rs3212986 had a worse DRC to the environmental carcinogen BPDE compared with CC genotype.

## 4 DISCUSSION

In present years, it has become increasingly clear that individuals’ susceptibility to the risk of carcinogenesis is highly related to interdifferent DRC. Many epidemiological investigations have demonstrated the relationship between polymorphisms with DNA repair genes and cancer risk.^26^, ^27^, ^28^ Although it was reported that SNPs in 3ʹUTR or haplotypes have relevance to the risk of several cancers, the conclusions were still quite controversial. The present study focuses on the association between lung cancer risk and 3ʹUTR polymorphisms of DNA repair genes based on a case‐control study.

*ERCC1* is a lead enzyme for a well functional NER, which can remove DNA damage in the global genome.^29^ Some *ERCC1* SNPs alert DNA repair capacity and therefore are considered as susceptibility biomarkers in predicting the cancer risk including NSCLC.^13^, ^30^, ^31^, ^32^, ^33^, ^34^ It was revealed that the minor allele of *ERCC1* rs3212986 may be a risk factor to alter DNA repair capacity, enhance genetic susceptibility to lung cancer,^30^, ^31^ shorten the carriers’ overall survival, and decrease the activity of platinum in advanced NSCLC.^32^, ^33^, ^34^ In this case‐control study, compared with *ERCC1* rs3212986 CC, AA genotype was shown to be a high‐risk factor for NSCLC (OR = 3.246; 95%CI: 1.375‐7.663). In agreement with this result, minor A allele of rs3212986 could also reflect a linkage with an increasing risk of NSCLC in haplotype analysis on chromosome 19. Besides hereditary factors, other factors such as gender, age, and smoking status also correlated with the formation of DNA adducts leading to distinct cancer susceptibility. Our stratified analysis showed that, compared with each wild type, AA genotype of *ERCC1* rs3212986 increased the risk of NSCLC in male population (OR = 3.246; 95% CI: 1.375‐7.663), meanwhile CC genotype of *OGG1* rs1052133 in female ones(OR = 2.588; 95% CI: 1.035‐6.474). *OGG1* is a key enzyme of BER to identify and excise 8‐oxoG lesions that induced transversions from G:C to T:A.^35^, ^36^, ^37^ Similarly, Liu et al^14^ observed that C allele site of *OGG1* rs1052133, jointed effects with a smoking habit, exerted an adverse influence on lung cancer risk in Asia. Moreover, the analysis after age stratification (Age < 60) indicated that AC genotype of *ERCC1* rs735482 had a protective role for lung cancer (OR = 0.560, 95%CI: 0.336‐0.934). However, Jones et al^38^ found no association between *ERCC1* rs735482 and lung cancer susceptibility in Caucasians. The inconsistent findings were possibly due to ethnic differences and limiting sample size. Furthermore, we found that the A allele (AA and CA + AA genotypes) of *ERCC1* rs3212986 also exhibited an enhanced risk to develop lung cancer in smokers only (*P* < 0.05), but not in never smokers. As we well known, gene‐smoking interactions have associations with the risk of lung cancer. Zhou et al^30^ consistently showed that the AA genotype of *ERCC1* rs3212986 polymorphism (or *ERCC1* haplotypes) had significant interaction between cumulative cigarette smoking and lung cancer risk. No significant association was observed between *ERCC1* rs2336219, *MLH3* rs108621, *PPP1R13L* rs6966, or *CD3EAP* rs1007616 polymorphisms and the risk of lung cancer.

**Figure 6:**
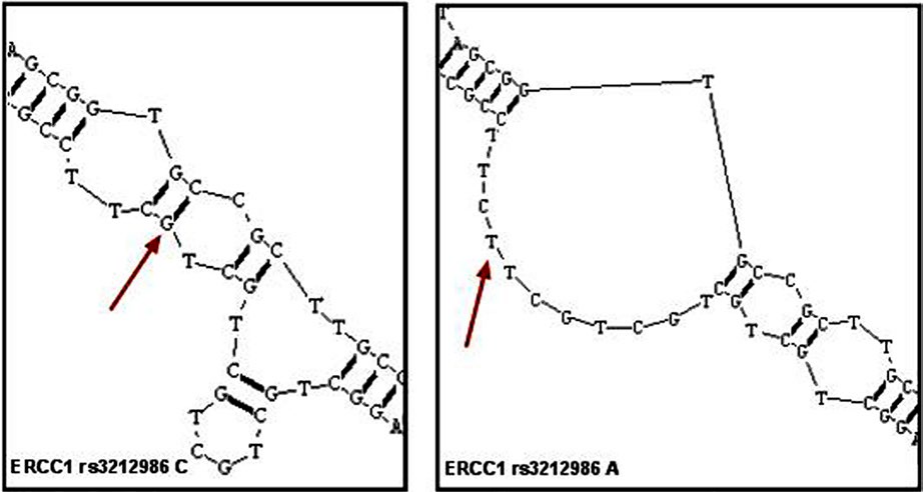
Rs3212986 variations might change ERCC1 mRNA structure and the stem‐loop structure formed by its upstream sequence would be unfolded. The RNA secondary structure was predicted by RNA structure Software. Only the most stable secondary structures with the lowest free energy are depicted

A potentially intriguing aspect of *ERCC1* rs3212986 polymorphism is that this SNP is also located in the coding region of *CD3EAP*.^23^ Our previous research using cultured lymphocytes from healthy population found that the minor A allele of *ERCC1* rs3212986 in genotypes and haplotypes remarkably increased BPDE‐induced DNA adducts, and AA homozygous cells also showed a reduced *CD3EAP* mRNA level in comparison with CC individuals after BPDE exposure.^17^ In this study, there was a strong LD between candidate SNPs of *ERCC1* and *CD3EAP*. And high NSCLC risk was observed within the haplotype blocks embracing A allele of *ERCC1* rs3212986. To analyze how rs3212986 polymorphism involved in DNA repair by affecting *ERCC1* or *CD3EAP*,* ERCC1*(CDS+3′UTR) cDNA, or *CD3EAP*(CDS) cDNA clones containing different genotypes of rs3212986 was introduced into human cell lines to magnify and illuminate this affection by inducing BPDE‐DNA adducts. We found that AA genotype was associated with lower cell survival rate, which may be representative for a less effective DNA repair and a higher level of DNA damage. In order to compare the DRC of damage caused by BPDE treatment in different transfected cells, DNA damage induced by BPDE‐DNA adduct was intuitively detected by γH2AX immunofluorescence and the modified comet assay. As a result, reduced DRC was observed in 293T^ERCC1(AA)^ and 16HBE^ERCC1(AA)^, whereas no significant difference in DRC was observed in 293T^CD3EAP(CC)^/16HBE^CD3EAP(CC)^ and 293T^CD3EAP(AA)^/16HBE^CD3EAP(AA)^, which suggested that the variant genotypes of rs3212986 modulated DNA repair efficiency via modifying 3ʹUTR of *ERCC1*, but not *CD3EAP*. Previous investigations have been reported that there was high LD between *ERCC1* rs3212986 and other polymorphisms of gene or polymorphisms of other adjacent genes on 19q13 such as *XRCC1* (X‐ray repair complementing defective repair in Chinese hamster cells 1) or *ERCC2/XPD* (excision repair cross‐complementation group 2/xeroderma pigmentosum D) resulting in an increased risk to develop lung cancer,^39^, ^40^ which may affect the mRNA stability of *ERCC1* and therefore be associated with a lower DNA repair efficiency.^41^ In addition, we predicted *ERCC1* mRNA secondary structure between two genotypes of rs3212986 using bioinformatics software. The expected results were showed that genetic variants in rs3212986 may play a critical effect on DRC by altering the folded stem‐loop structure consisting of six repeats of "GCT," which can impact 18‐base sequence in *ERCC1* 3ʹUTR (Figure 6). Moreover, enzyme expression may be regulated by miRNAs which can also be affected by polymorphism in target complementary sequence. As either oncogenes or tumor suppressors, miRNAs might have a synergistic effect of SNPs on individual DRC in relation to cancerous susceptibility.^42^, ^43^ Since located in the *ERCC1* 3′UTR, rs3212986 was likely involved in post‐transcriptional regulation of *ERCC1* by the specific binding of miRNAs as miR‐15a.^15^ Taken together the above results, although rs3213986 polymorphism mapping in overlapping genes *ERCC1* and *CD3EAP* on chromosome 19 was involved in expression of two genes, the major effect of DRC was driven by regulating *ERCC1* in 3′UTR, given the higher LD, unstable secondary structure, and posttranscriptional regulation of binding miRNAs. On the other hand, although we did not see significant difference in DNA repair efficiency between *CD3EAP* transfected cells with different rs3212986 genotypes after exposure to BPDE, we will not deny the importance of *CD3EAP* in DNA repair. It was noteworthy to find in the present study, contrasted to the empty vector transfected cells, the survival rates can be increased in overexpressed *CD3EAP* cells after the exposure of various BPDE concentrations. *CD3EAP* may probably be related to cell proliferation involving in the RNA polymerase I transcription complex.^18^, ^44^ In another previous study, we confirmed that co‐expression patterns were consisted in overlapping genes as *ERCC1*,* CD3EAP,* and *PPP1R13L.* Particularly, there was a significant association between *CD3EAP* exon 3 and *ERCC1* exon 11, while *CD3EAP* exon 1 and *PPP1R13L* exon 1.^45^ The potential influence of *CD3EAP* expression was in response to the regulation of genetic networks known to be associated with DRC and lung cancer risk.

In conclusion, as one part of our series complementary assays for lung cancer risk assessment project, the present study demonstrated that *ERCC1* C8092A (rs3213986) in 3ʹUTR of *ERCC1* was associated with an increased risk of lung cancer, especially in smoking population, and therefore could be used as a valuable tumor marker. Although it was also located within the ORF of *CD3EAP* gene, its effects on the DRC toward BPDE induced DNA damage mainly via modulating ERCC1 expression, but not *CD3EAP*. However, further studies involving more polymorphisms, conditional gene knockout model cell lines, and a larger sample size are still needed to clarify the detailed function of SNPs in 3ʹUTR.
