# Supplementary material for: Rs3212986 polymorphism, a possible biomarker to predict smoking‐related lung cancer, alters DNA repair capacity via regulating ERCC1 expression
Source: Cancer Med. 2018 Nov 19;7(12):6317–30. doi: 10.1002/cam4.1842 (PMC6308093; doi:10.1002/cam4.1842)
Supplement: Supplementary file 1 [file CAM4-7-6317-s001.docx]

**Table S1** The minor allele frequency (MAF) of *ERCC1, OGG1, MLH3, PPP1R13L* and *CD3EAP*

| Genes | SNP | Variation | MAF |
| --- | --- | --- | --- |
| *ERCC1* | rs735482 | A/C | 0.378 |
| *ERCC1* | rs2336219 | G/A | 0.378 |
| *ERCC1* | rs3212986 | C/A | 0.329 |
| *MLH3* | rs108621 | T/C | 0.232 |
| *OGG1* | rs1052133 | G/C | 0.45 |
| *PPP1R13L* | rs6966 | T/A | 0.386 |
| *CD3EAP* | rs1007616 | C/T | 0.398 |

**Table S2** General information of study population.

| Variables | Cases | Controls | *P*^a^ |
| --- | --- | --- | --- |
|  | n(％) | n(％) |  |
| Total | 300 (100.00) | 300(100.00) |  |
| Age(y) |  |  | 0.411 |
| ≤50 | 41(13.67) | 43(14.33) |  |
| 51-60  61-70 | 122(40.67)  97(32.33) | 123(41.00)  82(27.33) |  |
| ＞70 | 40(13.33) | 52(17.33) |  |
| Gender |  |  | 1.000 |
| Male | 188(62.67) | 188(62.67) |  |
| Female | 112(37.33) | 112(37.33) |  |

a: Pearson χ2 test for difference in distributions between the case and control groups.

**Table S3** The basic information of certain smoking population in case and control

| Variables | Cases | Controls | | P^a^ |
| --- | --- | --- | --- | --- |
|  | n(％) | n(％) | |  |
| Total | 186 (100.00) | 186(100.00) | |  |
| Age(y) |  | |  | 0.590 |
| ≤50 | 29(15.59) | 28(15.05) | |  |
| 51-60  61-70 | 77(41.40)  60(32.26) | 73(39.25)  56(30.11) | |  |
| ＞70 | 20(10.75) | 29(15.59) | |  |
| Gender |  | |  | 1.000 |
| Male | 102(54.84) | 102(54.84) | |  |
| Female | 84(45.16) | 84(45.16) | |  |
| Smoking |  |  | | 1.000 |
| Yes | 81(43.55) | 81(43.55) | |  |
| No | 105(56.45) | 105(56.45) | |  |
| Alochol consumption |  |  | | 1.000 |
| Yes | 64(34.41) | 64(34.41) | |  |
| No | 122(65.59) | 122(65.59) | |  |

a: Pearson χ2 test for difference in distributions between the case and control groups.
